# Different Modes of Gene Duplication Show Divergent Evolutionary Patterns and Contribute Differently to the Expansion of Gene Families Involved in Important Fruit Traits in Pear (*Pyrus bretschneideri*)

**DOI:** 10.3389/fpls.2018.00161

**Published:** 2018-02-13

**Authors:** Xin Qiao, Hao Yin, Leiting Li, Runze Wang, Juyou Wu, Jun Wu, Shaoling Zhang

**Affiliations:** State Key Laboratory of Crop Genetics and Germplasm Enhancement, Centre of Pear Engineering Technology Research, Nanjing Agricultural University, Nanjing, China

**Keywords:** duplicate genes, evolution, gene family, metabolism pathways, fruit traits, pear

## Abstract

Pear is an important fruit crop of the Rosaceae family and has experienced two rounds of ancient whole-genome duplications (WGDs). However, whether different types of gene duplications evolved differently after duplication remains unclear in the pear genome. In this study, we identified the different modes of gene duplication in pear. Duplicate genes derived from WGD, tandem, proximal, retrotransposed, DNA-based transposed or dispersed duplications differ in genomic distribution, gene features, selection pressure, expression divergence, regulatory divergence and biological roles. Widespread sequence, expression and regulatory divergence have occurred between duplicate genes over the 30–45 million years of evolution after the recent genome duplication in pear. The retrotransposed genes show relatively higher expression and regulatory divergence than other gene duplication modes. In contrast, WGD genes underwent a slower sequence divergence and may be influenced by abundant gene conversion events. Moreover, the different classes of duplicate genes exhibited biased functional roles. We also investigated the evolution and expansion patterns of the gene families involved in sugar and organic acid metabolism pathways, which are closely related to the fruit quality and taste in pear. Single-gene duplications largely account for the extensive expansion of gene families involved in the sorbitol metabolism pathway in pear. Gene family expansion was also detected in the sucrose metabolism pathway and tricarboxylic acid cycle pathways. Thus, this study provides insights into the evolutionary fates of duplicated genes.

## Introduction

Gene duplication has long been regarded as an important evolutionary force that provides abundant raw materials for genetic novelty, morphological diversity and speciation (Ohno, 1970; Zhang, 2003; Flagel and Wendel, 2009; Panchy et al., 2016). Gene duplication can occur by several mechanisms, including whole-genome duplication (WGD) and single gene duplication. Single gene duplication includes four types, tandem (TD), proximal (PD), retrotransposed (RD), DNA-transposed (DD) and dispersed duplication (DSD) (Freeling, 2009; Hahn, 2009; Wang et al., 2012b). WGD (also known as polyploidization) duplicates all of the nuclear genes of an organism at once and generates a huge number of duplicated genes. Paleopolyploidization is rampant in the plant kingdom and is the dominant feature of plant genome evolution but not the evolution of animals and fungi (Moghe and Shiu, 2014; Michael and VanBuren, 2015; Wendel, 2015; Salman-Minkov et al., 2016). In addition to WGD, single gene duplication is also prevalent in plant genomes over long evolutionary time periods (Freeling, 2009; Wang Y. et al., 2011; Wang et al., 2012b). However, the gene loss after gene or genome duplication is very common in plant genomes (Lynch and Conery, 2000).

Tandem duplications often occur as a result of unequal crossing over and are often followed by inversion events (Freeling, 2009; Hahn, 2009). The proximal gene pair comprises two gene copies that are closely located on the chromosome but separated by a few genes (Wang et al., 2012b). Two contiguous gene duplicates that originated from ancient tandem duplication events can be disrupted by inserting other genes (Freeling et al., 2008), which is assumed to be a source of proximal duplicates. In addition, localized transposon activities can result in the proximal duplications (Zhao et al., 1998). Transposed duplication events can take place through DNA-based or RNA-based transposition (or retrotransposition) in which the duplicated gene is relocated to a new chromosomal position (Freeling, 2009; Hahn, 2009; Wang et al., 2012b). However, the mechanism underlying the abundance of dispersed duplicated genes remains unclear. Because of the various genetic mechanisms for generating different modes of gene duplications, we can speculate that different types of gene duplications may evolve along distinct evolutionary trajectories, and may have been retained in a biased manner over long evolutionary time periods.

The preservation of duplicate genes can be attributed to the interactions of multiple factors, such as gene features, gene expression level, alternative splicing and protein–protein interactions (Du et al., 2012; Guo et al., 2012; Grishkevich and Yanai, 2014; McGrath et al., 2014; Diss et al., 2017). The evolutionary rate, structural complexity, and GC3 content may be intensely correlated with the retention of WGD-derived duplicated genes (Jiang et al., 2013). The expression divergence between duplicated genes occurred ubiquitously after gene duplication in plant genomes (Blanc and Wolfe, 2004; Renny-Byfield et al., 2014). A positive correlation between structural divergence and gene expression divergence has been observed in Arabidopsis (Wang et al., 2013b). Following gene duplication, the divergence of the promoter sequence between duplicated genes may lead to their expression divergence (Zhang, 2003; Hahn, 2009). The frequent gain and loss of *cis*-regulatory elements contained in promoters between parent and daughter genes occurred shortly after gene duplication, resulting in subfunctionalization (Force et al., 1999; Lynch and Force, 2000) and neofunctionalization (He and Zhang, 2005; Arsovski et al., 2015). Another important model underlying duplicated gene retention following WGD is the gene dosage balance model (Birchler and Veitia, 2007). This model states that those duplicated genes that are dosage-sensitive or frequently interact with other genes tend to be retained because the loss of one of the duplicates causes dosage imbalances and decreases fitness. Many other evolutionary models have also been proposed to elucidate the mechanisms underlying the short- and long-term retention of duplicated genes (Freeling, 2009; Conant et al., 2014; Panchy et al., 2016), including absolute dosage constraints (Bekaert et al., 2011; Hudson et al., 2011; Conant et al., 2014), dosage subfunctionalization (Gout and Lynch, 2015), and compensatory drift model (Thompson et al., 2016). However, the relationships among structural, expression and regulatory divergences between duplicated genes are not well understood. What factors maintain the genetic redundancy over long time periods are still controversial.

In this study, we first aimed to build a standard procedure to identify different modes of duplicated genes, including genes derived from WGD, TD, PD, RD, DD, and DSD. Second, we attempted to explore the relationship among sequence, expression and regulatory divergence. Third, we further addressed whether different modes of duplicated genes evolved toward biased functional roles. In addition, the contribution of gene duplication to biological innovation was evaluated by investigating the expansion patterns of gene families involved in key fruit traits.

## Materials and Methods

### Data Collection

Chinese white pear (*Pyrus bretschneideri*) genome sequences and annotation files were downloaded from the Pear genome project^1^ (Wu et al., 2013). Chinese plum (*Prunus mume*) genome sequences and annotation information were downloaded from the *Prunus mume* Genome Project^2^ (Zhang et al., 2012). Apple (*Malus × domestica*) whole genome data was obtained from GDR^3^ (Jung et al., 2014). The other 32 plant genome data sets were downloaded from Phytozome v9.1^4^ (Goodstein et al., 2012).

### Identification of Different Modes of Duplicated Genes

The MCScanX software package (Wang et al., 2012a) was used to identify the WGDs/segmental, tandem and proximal duplications in the pear genome. Genes within the pear genome were classified as singletons, dispersed, proximal, tandem and segmental/WGD duplicates using the MCScanX package. First, an all-vs.-all local BLASTP algorithm-based search was performed for all protein sequences from the pear genome (*E* < 1 e^-5^, top five matches and m8 format output). Second, *duplicate gene classifier*, the core program of MCScanX, was executed using the BLASTP output and annotation file as the input files. The modes of gene duplication were determined using the algorithm within MCScanX according to the following procedure: all genes were initially ranked according to their order along chromosomes and were labeled as singletons. Gene pairs within BLASTP hits were then evaluated. If the genes had BLASTP hits to other genes, then they were re-labeled as dispersed duplicates. If the two genes in a BLASTP hit had a difference of gene rank < 20 (configurable), then they were re-labeled as proximal duplicates. If the two genes had a difference of gene rank = 1, then they were re-labeled as tandem duplicates. Finally, the anchor genes in collinear blocks were re-labeled as WGD/segmental duplicates (Wang et al., 2012a). Duplicated genes were assigned to a unique pattern according to the order of priority: WGD/segmental > tandem > proximal (Wang et al., 2012a).

Furthermore, transposed duplications, including RNA-based transposed duplications (RDs) and DNA-based transposed duplications (DDs) were identified. A transposed duplicate pairs must be meet the following criteria: one gene existed in its ancestral locus, and the other was located in a non-ancestral locus (Wang Y. et al., 2011). Therefore, ancestral gene locations were first discerned by synteny aligning. The synteny analyses between pear and 34 other plant genomes were conducted locally using a method similar to that developed for the Plant Genome Duplication Database (PGDD)^5^ (Tang et al., 2008a; Lee et al., 2013). Then, all syntenic blocks between pear and the 34 other species mentioned earlier were identified. Finally, genes located in these syntenic blocks in pear were deemed to be ancestral loci. To search transposed duplications, WGD/segmental, tandem and proximal duplicate pairs were excluded from the BLASTP results. The BLASTP hits containing an ancestral and a novel locus were defined as transposed duplications. If a pair of transposed duplicated genes comprised an ancestral gene with more than two exons and a novel transposed copy without an intron, then this pair was inferred to be derived from RNA-based transposition (retrotransposition). If both genes in a transposed duplicated pair had a single exon, the pair of duplicates was removed temporarily. The other remaining pairs of transposed duplicated genes were inferred to have originated from DNA-based transposition (Wang Y. et al., 2011). In the present study, because multiple ancestral loci may be found for a transposed duplicate, the ancestral locus with the highest similarity was identified as the parental duplicate (Wang et al., 2013c).

After excluding WGD/segmental, tandem, proximal, retrotransposed and DNA-based transposed duplications, the remaining duplicated gene pairs from the BLASTP output were defined as DSDs. After all duplicated pairs were classified into different patterns, each duplicated gene was assigned to a unique mode. The priority of duplicated genes was as follows: WGD > tandem > proximal > retrotransposed > DNA-based transposed > dispersed.

### Calculation of Non-synonymous (*K*_a_) and Synonymous (*K*_s_) Substitution Rates and *K*_a_/*K*_s_ Ratios

The valid duplicate gene pairs originated from different duplication modes were used to calculate the *K*_a_ and *K*_s_ substitution rates. *K*_a_*K*_s__Calculator 2.0 was used to estimate *K*_a_ and *K*_s_ values, and the *K*_a_/*K*_s_ ratios (Wang et al., 2010). We adopted a model-averaged method to measure the *K*_a_, *K*_s_, and *K*_a_/*K*_s_. This method averages parameters across 14 candidate models (Zhang et al., 2006; Wang et al., 2010, 2013a). The parameters configuration used was as described in the *K*_a_*K*_s__Calculator 2.0 software package manual.

### RNA-seq Data and Quantification

The raw RNA-seq reads for Chinese white pear (‘Dangshansuli’) were downloaded from NCBI SRA^6^. The information regarding the RNA-seq samples used in this study can be retrieved from Supplementary Table 6. The raw reads were filtered using Trimmomatic (version 0.36) by performing the following trimming steps: (1) removing adapter sequences; (2) excluding leading or trailing low quality or N bases (below quality 15); (3) cutting sequences in which the average quality per base drops is below 15 when scanned the read with a 4-base wide sliding window; and (4) discarding reads shorter than 55 and 36 bp for paired-end and single-end reads, respectively (Bolger et al., 2014; Kagale et al., 2016). The high-quality clean reads were adopted in the downstream analysis. The abundance levels of transcripts from RNA-seq data were estimated using Kallisto (Bray et al., 2016). The reference transcripts obtained from pear genome annotation files were used to construct a Kallisto index. Then, the Kallisto quantification algorithm was performed with default parameters (for single-ends, -l 200 -s 20) to process single-end or paired-end reads. The output included the normalized count estimates and TPM values for each transcript. The TPM value was used as the measure of gene expression levels in different tissues and developmental stages. Furthermore, we extracted all of the intergenic regions at the whole-genome level for pear, and then we quantified the expression abundance levels for intergenic sequences using the same procedure and RNA-seq reads that were used for the above exonic regions. We used the mean value (0.715) of the medians (the 50th percentile) obtained from the TPM distributions for intergenic sequences in different tissues and developmental stages as the threshold of expression (**Figure 4A**). Therefore, any gene with a TPM > 0.715 was considered expressed in pear.

### Estimating Expression Divergence

Here, we only used those duplicated pairs in which both gene copies were expressed in at least one tissue (Makova and Li, 2003; Wang et al., 2016). The Pearson correlation coefficients (*r*) between the expression profiles of each gene pair were computed using the “Scipy” module in Python. Then, we established a cutoff *r*-value below which two duplicate genes were considered divergent in expression. We randomly selected 10,000 gene pairs and computed *r*-values for their expression profiles. In total, 95% of the *r*-values for these random pairs were *r* < 0.89; therefore, the gene pairs with *r* ≥ 0.89 were assumed to have significantly conserved expression levels at α = 0.05. In the present study, the gene pairs with *r* < 0.89 were considered to have diverged in expression.

### Collecting Promoter Sequences and Estimating Regulatory Divergence

As the putative promoter sequence, 1000 bp upstream of the transcriptional start site for each gene was extracted using BEDTools (Quinlan and Hall, 2010). Then, we used SharMot (-l 16) to estimate the promoter-sequence divergence (*d*_SM_; shared-motif divergence) for each gene pair (Castillo-Davis et al., 2004; Farre and Alba, 2010). The local similarity of promoter sequence between two duplicated genes was measured by s_LS_ = 1 -*d_SM_*. We randomly selected 10,000 gene pairs and computed their *s*_LS_ values. In total, 95% of the *s*_LS_ values for these random pairs were *s*_LS_ < 0.60; therefore, gene pairs with *s*_LS_ ≥ 0.60 were assumed to have significantly conserved promoter regions at α = 0.05. Because random gene pairs have unrelated promoters and a lower *s*_LS_ value, any duplicated gene pairs with *s*_LS_ < 0.60 was considered to have diverged in the promoter region.

### Detecting Gene Conversion between Duplicate Genes

In this study, we investigated the whole-gene conversion for each gene pair generated by different modes of gene duplication in pear. First, we determined the homologous gene quartets, comprised of two paralogs in pear and their respective orthologs in apple (outgroup species). Then, we compared the gene similarity or tree topology between homologs in quartets by estimating their *K*_s_ value. Bootstrap tests of 1000 repetitive random samples was performed to evaluate the significance of putative gene conversions. Because the genome duplication occurred before species divergence between pear and apple, we hypothesized that the pear-apple orthologs would be more similar to one another than to their respective paralogs in each species. However, if the paralogs had experienced gene conversion after speciation, we would observe they would be more similar to each other than to their respective orthologs (Wang et al., 2007, 2009; Wang X. et al., 2011).

### Pfam Domain Analysis

The HMM profile database-Pfam-A.hmm was downloaded from the Pfam protein families database (version 27.0^7^) (Finn et al., 2014). Then, we used *hmmpress* and Pfam-A.hmm to construct binary compressed data files for *hmmscan* (Eddy, 2011). Lastly, *hmmscan* was used to search for conserved domains in the annotated proteins with *E* < 1 e^-5^. We studied all of the domains detected in WGD, TD, PD, RD, DD, and DSD proteins. For each domain, we calculated the percentage of the domains represented in the different duplication modes of proteins or among the total proteins.

### Gene Ontology (GO) Enrichment Analysis

The GO annotation for pear genes was obtained from the pear genome project^8^ (Wu et al., 2013). The three top GO categories: molecular function (MF), biological process (BP), and cellular component (CP) were analyzed (Ashburner et al., 2000). The enriched GO slim terms were determined using the program package GOATOOLS (Tang et al., 2015). The *P*-values used to evaluate the significant enrichment of certain GO terms were calculated based on Fisher’s exact test and corrected using the false discovery rate (FDR) test correction method (FDR implementation using resampling). Finally, we used a corrected *P*-value < 0.05 as the significance cut-off to determine the significant over-representation of certain GO terms.

### Identification of Gene Families Involved in Sugar and Organic acid Metabolism Pathways

The referred IDs for the sugar- and acid-related metabolism genes in Arabidopsis were obtained from previous studies (Shangguan et al., 2014; Shangguan et al., 2015). The corresponding protein sequences of Arabidopsis were downloaded from Phytozome v11^9^. Then, we performed a local BLASTP algorithm-based search (*E* < 1 e^-10^) against the pear whole-genome protein sequences using the protein sequences of Arabidopsis as queries. Finally, the gene family members involved in the sugar and organic acid metabolism pathways were determined in pear.

## Results

### Genome-Wide Identification of Different Modes of Gene Duplication

The local all-vs.-all BLASTP algorithm-based search was conducted using whole-genome protein sequences (42,341) to search populations of potential duplicated gene pairs. The gene duplication population contained 38,593 genes (91% of all genes) (Supplementary Table 1). We attempted to search the six modes of duplicated gene pairs, respectively, derived from WGD, TD, PD, RD, DD, and DSD. The MCScanX package was used to detect WGD- and TD-derived gene pairs, while the other modes of duplicated gene pairs were determined according to the procedures described in the Methods section. As a result, we identified 13,638 and 2626 gene pairs derived from WGD and TD, respectively (Supplementary Table 2). Additionally, 1288 gene pairs derived from PD were further identified according to the chromosomal interval (10 or fewer genes) between two genes from a BLASTP hit. After removing WGD-, TD-, and PD-derived pairs from the population of gene duplications (or BLASTP hits), we continued to search for RD-, DD-, and DSD-derived gene pairs. Finally, a total of 217 RD-, 1188 DD-, and 18945 DSD-derived pairs were identified.

### Genomic Distribution and Gene Features of Different Modes of Duplicated Genes

**FIGURE 1:**
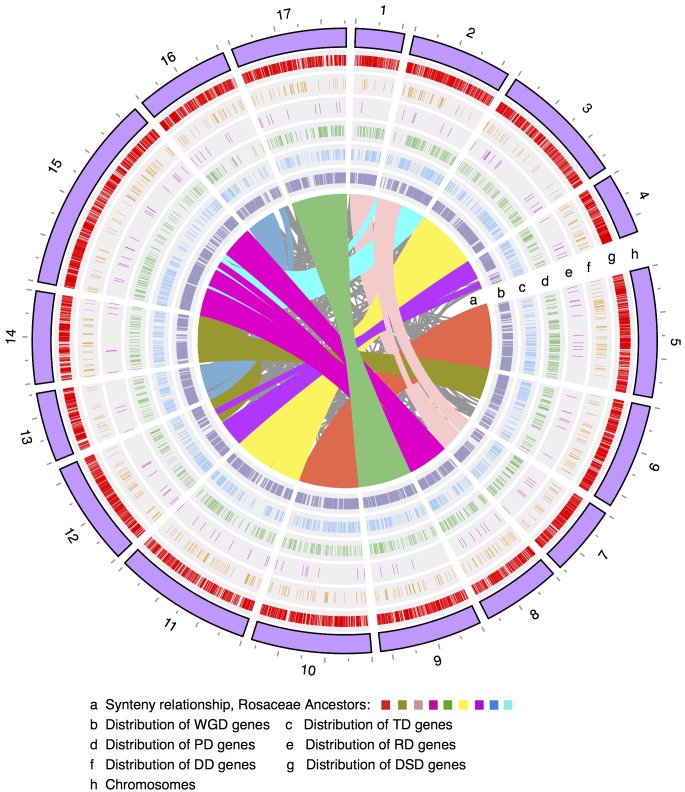
The chromosomal distribution of different modes of duplicated genes. WGD, whole-genome duplication; TD, tandem duplication; PD, proximal duplication; RD, retrotransposed duplication; DD, DNA-transposed duplication; DSD, dispersed duplication.

The number of WGD-derived duplicated genes on each of the 17 pear chromosomes ranged from 415 (415/42,341 = 1.0%, Chr4) to 2082 (4.9%, Chr15), while TDs ranged from 98 (0.2%, Chr1) to 262 (0.6%, Chr15), PDs ranged from 62 (0.1%, Chr13) to 211 (0.5%, Chr5), transposed duplicates (RD and DD) ranged from 9 (0.02%, Chr1) to 78 (0.2%, Chr15), and DSDs ranged from 233 (0.6%, Chr1) to 1080 (2.6%, Chr15) (**Figure 1** and Supplementary Figure 1). Moreover, the density levels of different modes of duplicated genes fluctuated greatly along each chromosome (Supplementary Figure 2). The high density of WGD-genes was located on the chromosomal arm region, resulting in a ‘V’-type distribution. A similar trend was observed in the genomic distributions of TD-, PD-, RD-, and DD-derived genes. However, the density levels of the DSD-genes in the pericentromeric or chromosomal arm regions are similar.

Furthermore, Pearson’s correlation coefficient (*r*) was used to measure the correlation of the genomic density between any two modes of duplicated genes. Some chromosomal regions with low frequency levels of WGD-derived genes often showed high frequency levels of DSD-derived genes (**Figure 1** and Supplementary Figures 2, 3). Indeed, a negative correlation was observed for the genomic density between WGD- and DSD-derived genes on 14 out of 17 pear chromosomes, and significant negative correlations were found on Chr1 (*r* = -0.65, *P*-value = 0.001), Chr4 (*r* = -0.040, *P*-value = 0.043), Chr7 (*r* = -0.60, *P*-value = 0.015) (Supplementary Table 3). Moreover, we found positive correlations for the genomic density between WGD- and TD- or PD-derived genes. In particular, the significant positive correlation between the density levels of WGD- and TD-derived genes was detected on 10 out of 17 chromosomes. In addition, the distributions of TD- and PD-derived genes overlapped to some extent on each chromosome. A positive correlation was found for the genomic density between TD- and PD-derived genes on 15 out of 17 chromosomes. Additionally, a significant positive correlation was found on the following nine chromosomes: Chr2 (*r* = 0.49, *P*-value = 0.016), Chr5 (*r* = 0.60, *P*-value = 0.001), Chr6 (*r* = 0.47, *P*-value = 0.021), Chr8 (*r* = 0.50, *P*-value = 0.039), Chr9 (*r* = 0.58, *P*-value = 0.003), Chr11 (*r* = 0.47, *P*-value = 0.008), Chr14 (*r* = 0.46, *P*-value = 0.036), Chr15 (*r* = 0.050, *P*-value = 0.015), and Chr17 (*r* = 0.67, *P*-value = 0.0002).

In addition, we investigated the gene features of different modes of duplicated genes, including the GC content, GC3 content, average exon length and coding-region length (Supplementary Figure 4). The RD-derived genes exhibited relatively higher GC and GC3 contents than other modes of duplicated genes. Moreover, the RD-derived genes showed a strong trend to longer average exon length and shorter coding-region lengths. In contrast, the DD-derived genes had shorter average exon lengths and longer coding-region lengths, suggesting that these genes possessed more exons. However, WGD-, TD-, PD-, and DSD-derived genes presented similar gene features.

### Selection Pressure Acting on the Different Modes of Gene Duplication

**FIGURE 2:**
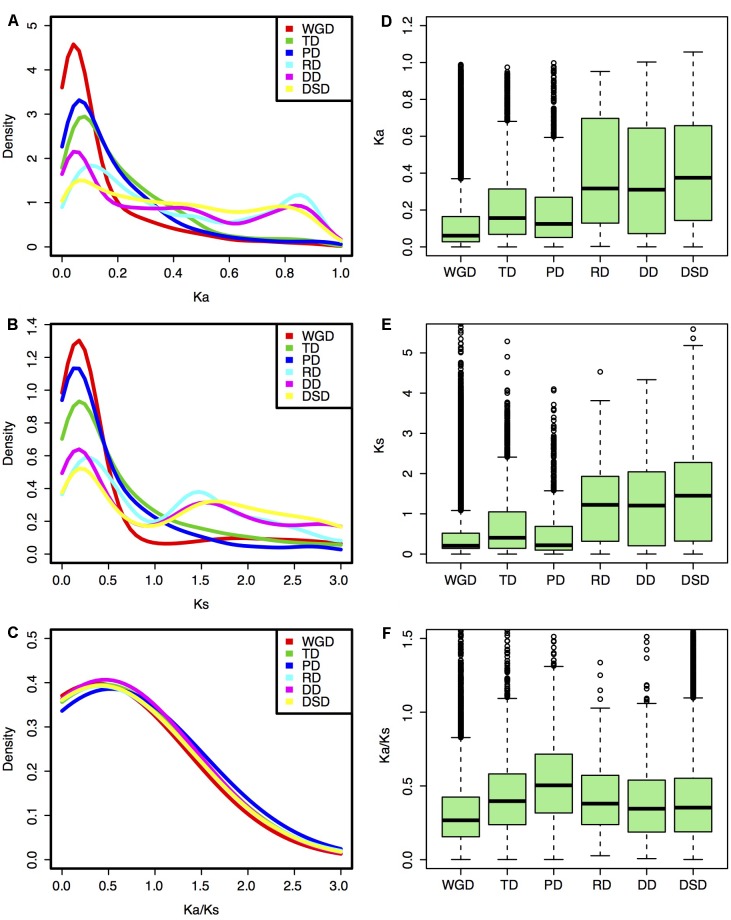
Evolutionary patterns of gene pairs duplicated by different modes in pear. **(A,D)** *K*_a_ distributions; **(B,E)** *K*_s_ distributions; **(C,F)** *K*_a_/*K*_s_ ratio distribution. **(A–C)** Density plot; **(D–F)** Box plot.

The *K*_a_, *K*_s_, and *K*_a_/*K*_s_ values were computed for each gene pair from different modes of gene duplication. Different gene duplication modes exhibited divergent *K*_a_ and *K*_s_ distributions. Two peaks (∼0.05 and ∼0.85) of *K*_a_ distributions for RD-, DD-, and DSD-derived pairs were observed, while only one peak (∼0.05) was observed for WGD-, TD-, and PD-derived pairs (**Figure 2A**). The boxplot further revealed that the RD-, DD-, and DSD-derived pairs had higher median of *K*_a_ distribution values than the other three modes, suggesting that they were more extensively mutated during the long evolutionary time period (**Figure 2D**). The *K*_s_ distributions for WGD-, RD-, DD-, and DSD-derived pairs presented two peaks (∼0.2 and ∼1.5), corresponding to the recent and ancient WGD events, respectively (**Figure 2B**). Moreover, the *K*_s_ peaks for WGD-, RD-, DD-, and DSD-derived pairs emerged at a similar *K*_s_ region or age, suggesting that the drastic genome fractionation or rearrangement occurred very shortly after WGD and resulted in extensive transposed and dispersed duplicates. In addition, the *K*_s_ peaks for TD- and PD-derived pairs occurred at smaller *K*_s_ values, and also overlapped with those of WGD-derived pairs (**Figure 2E**). Notably, the TD- and PD-derived pairs had higher *K*_a_/*K*_s_ ratios than the other modes (**Figures 2C,F**), indicating that these genes have been subjected to stronger selection pressures and may serve as good targets for neofunctionalization. However, the WGD-derived pairs possessed the smallest *K*_a_/*K*_s_ ratios compared with those of the other gene classes, implying that the surviving WGD-derived genes had undergone a more slow sequential or functional divergence for a long time periods.

**FIGURE 3:**
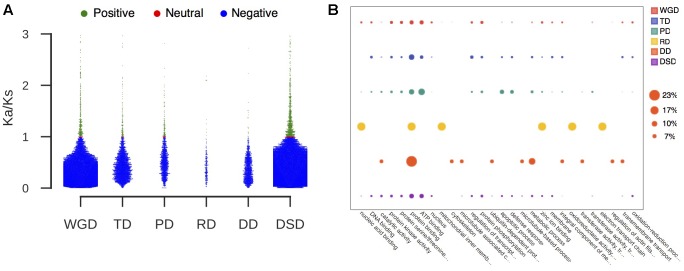
The characteristic of duplicated genes that experienced positive selection. **(A)** The proportion of duplicated gene pairs under different selection pressure. Green dot: positive selection; red dot: neutral selection; blue dot: negative selection. **(B)** GO analysis of duplicated genes that experienced positive selection. The larger circle indicates a higher frequency of occurrence of a GO term.

We further classified the duplicated gene pairs into three groups based on their different selection pressures (**Figure 3A**). Most of gene pairs had evolved under purifying selection (*K*_a_/*K*_s_ < 1). In contrast, rare gene pairs had evolved under neutral selection (*K*_a_/*K*_s_ = 1), and a small proportion of gene pairs had evolved under positive selection (*K*_a_/*K*_s_ > 1). The percentage of PD-derived pairs (7.8%) that was subjected to positive selection was highest among the different duplication modes, and less duplicated genes showed evidence of positive selection in WGD- or DD-derived pairs. Furthermore, we performed the GO analysis for those duplicated genes undergoing positive selection to explore their functional roles (**Figure 3B** and Supplementary Table 4). Protein binding (GO:0005515) was overrepresented in all modes of duplicate genes under positive selection. The WGD-, TD-, PD-, and DSD-derived genes that evolved under positive selection were also enriched in ATP binding (GO:0005524). In addition, a number of duplicated gene that underwent positive selection were involved in protein kinase activity (GO:0004672), protein serine/threonine kinase activity (GO:0004674), and protein phosphorylation (GO:0006468).

In addition, we investigated the whole-gene conversion events that occurred in different modes of duplicated gene pairs. RD- and DD-derived pairs were excluded in the following analysis because their homologous gene quartets were not identified. We found that 337 WGD-, 56 TD-, 29 PD-, and 39 DSD-derived pairs were influenced by gene conversion (Supplementary Table 5). Interestingly, most converted WGD-derived pairs were located within syntenic chromosome pairs, such as Chr 5 and Chr 10, and Chr 3 and Chr 11 (Supplementary Figure 5). The high frequency of gene conversion that occurred in WGD-derived pairs may partially account for their lower sequence divergence levels. The functional roles of converted gene pairs were further analyzed (Supplementary Figure 6). The duplicated gene pairs that underwent gene conversion were enriched in protein binding (GO:0005515) and ATP binding (GO:0005524). Additionally, apoptotic process (GO:0006915) and defense response (GO:0006952) were overrepresented in converted PD-derived pairs.

### Expression Divergence and Promoter Divergence Levels between Duplicated Genes

**FIGURE 4:**
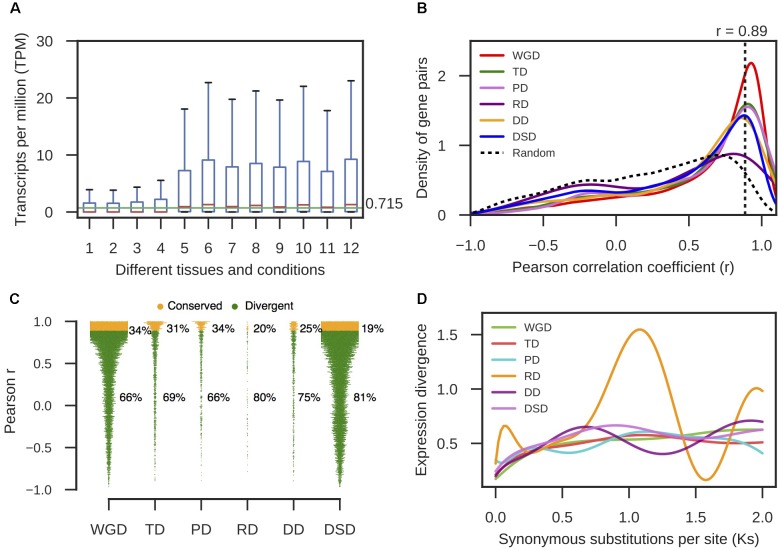
The expression divergence between duplicate genes in pear. **(A)** The distributions of TPM values in different tissues and conditions for intergenic sequences. The horizontal green line indicates the mean value of the medians in different boxplots. **(B)** The density distributions of Pearson’s correlation coefficient (*r*) between the expression profiles of two copies of different modes of duplicated pairs. The vertical dotted line indicates the 95% quantile in the *r*-values distribution for 10,000 random gene pairs. **(C)** The proportion of divergent and undifferentiated (or conserved) gene pairs in expression. **(D)** The dynamic expression divergence between duplicate genes with increasing *K*_s_ values.

RNA-seq data from different pear tissues and development stages were collected to comprehensively measure the expression divergence between duplicated genes (Supplementary Table 6). We adopted TPM > 0.715 as the threshold of expression for pear genes (see section “Materials and Methods” for details, **Figure 4A**). Here, we only analyzed the duplicated pairs in which both gene copies were expressed in at least one tissue or developmental stage. The *r*-value was calculated between the expression profiles of two copies of each gene pair, and 1-*r* was used to measure the expression divergence between duplicated genes. To determine the cutoff that indicated two gene copies of a pair had diverged in expression, we randomly selected 10,000 gene pairs and computed *r* between their expression profiles. Then, the 95% quantile in the distribution of *r*-values for random gene pairs was taken as the cutoff (*r* < 0.89) (**Figure 4B**). In total, 66% WGD-, 69% TD-, 66% PD-, 80% RD-, 75% DD-, and 81% DSD-derived pairs have diverged in expression (**Figure 4C**). RD-, DD-, and DSD-pairs experienced more drastic divergence in expression. Moreover, we investigated the dynamic process of expression divergence using *K*_s_ values for different modes of gene duplication in pear. We used the Python NumPy module to fit the smooth curve between expression divergence and *K*_s_ for each mode of duplicated gene pairs with 10 degrees of freedom (**Figure 4D**). When *K*_s_ < 0.5, the expression divergence of different modes of gene duplication gradually increased with increasing *K*_s_ values. The RD-derived pairs appear to have experienced more dramatic expression divergence than the other classes of duplicated genes. The abnormal curve for RD-derived pairs may be resulted from there being fewer of these pairs available RD pairs when fitting the curve between pearson r and *K*_s_ for RD-derived pairs using a smooth spline with 10 degrees of freedom (**Figure 4D**). Initially, 219 RD-derived pairs were identified in this study. After filtering the RD-derived pairs with abnormal or null r or *K*_s_ values, only 56 RD-derived pairs were reserved.

**FIGURE 5:**
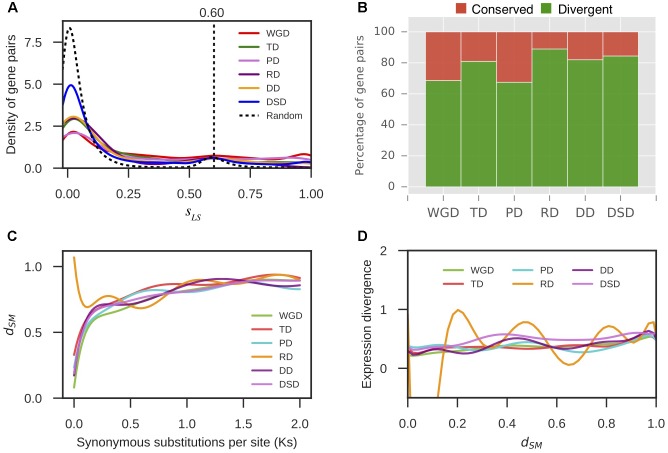
The promoter region divergence between duplicate genes in pear. **(A)** The density distribution of the shared-motif similarity in the promoter sequence (*s*_LS_) between two duplicated genes resulting from different modes of duplicated pairs. The vertical dotted line indicates the 95% quantile in the *s*_LS_ values distribution for 10,000 random gene pairs. **(B)** The proportion of divergent and conserved gene pairs in the promoter region. **(C)** The dynamic promoter divergence between duplicate genes with increasing *K*_s_ values. *d*_SM_ indicates the shared-motif divergence of the promoter. **(D)** The dynamic expression divergence between duplicated genes with increasing *d*_SM_ values.

Furthermore, we extracted 1000 bp upstream of the transcription start site for each gene as the putative promoter sequence. The local similarity level of promoter sequences between two gene copies of a gene pair was measured by *s*_LS_ (see section “Materials and Methods” for details). We randomly selected 10,000 gene pairs and computed their *s*_LS_ values. In total, 95% of the *s*_LS_ values for these random pairs were *s*_LS_ < 0.60; therefore, gene pairs with *s*_LS_ ≥ 0.60 were assumed to be significantly conserved in the promoter region at α = 0.05 (**Figure 5A**). Thus, the duplicated gene pairs with *s*_LS_ < 0.60 were considered to have diverged in the promoter region. Thus, 69% WGD-, 81% TD-, 67% PD-, 89% RD-, 82% DD-, and 84% DSD-derived pairs have diverged in their promoter regions (**Figure 5B**). The RD-, DD-, and DSD-derived pairs, which had undergone extensive expression divergence, had dramatically diverged in their promoter regions. Furthermore, the dynamic process of promoter divergence with *K*_s_ was dissected for different modes of gene duplication in pear. The smooth curve between promoter divergence and *K*_s_ was fitted with 10 degrees of freedom for each mode of duplicated gene pairs (**Figure 5C**). The promoter divergence appeared to increase exponentially with increasing *K*_s_ values, except for RD-derived pairs that showed exponential decreases with increasing *K*_s_ values at *K*_s_ < 0.5. In addition, the smooth curve between expression divergence and promoter divergence was fitted with 10 degrees of freedom for each mode of duplicated gene pairs. However, the promoter divergence between duplicated genes showed no significant correlation to expression divergence (**Figure 5D**).

**FIGURE 6:**
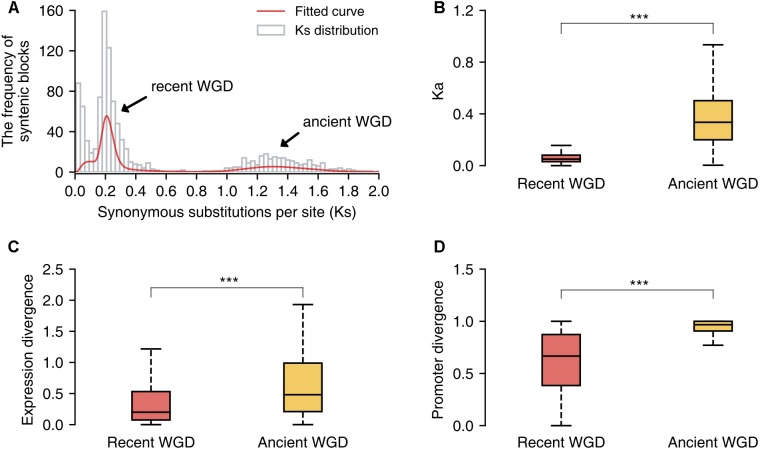
The comparison of sequence, expression and promoter divergence between duplicated genes derived from recent and ancient whole-genome duplications (WGD) events. **(A)** The distribution of *K*_s_ values of syntenic/paralogous blocks within the pear genome. **(B)** The comparison of sequence divergence between duplicated genes from two different WGD events. **(C)** The comparison of expression divergence between duplicate genes from two different WGD events. **(D)** The comparison of promoter divergence between duplicate genes from two different WGD events. Significant differences (Mann–Whitney *U* test): ^∗∗∗^*P* < 0.001.

Additionally, we identified the duplicated gene pairs retained from the recent and ancient WGD events in the pear genome and compared the patterns of divergence between these two sets of genes. First, we identified 1058 paralogous/syntenic chromosome blocks within pear genome, and then we calculated the mean *K*_s_ values for the gene pairs contained in each pair of duplicated blocks. Furthermore, two *K*_s_ peaks corresponding to the two WGD events were fitted from the *K*_s_ distribution by using mixture models with two components (Tang et al., 2008b) (**Figure 6A**). The duplicated gene pairs reserved from different WGD events were retrieved from those paralogous blocks with *K*_s_ = 0.15–0.25 (recent WGD) and *K*_s_ = 1.2–1.5 (ancient WGD), respectively. The non-synonymous substitution rates (*K*_a_) were used to measure the sequence divergence between duplicated genes. The gene duplicates derived from the ancient WGD event have experienced greater divergence than those from the recent WGD event (Mann–Whitney *U* test, *P*-value < 0.001) (**Figure 6B**). In parallel, gene pairs retained from the more ancient WGD showed greater expression and promoter divergence than those from the recent WGD (Mann–Whitney *U* test, *P*-value < 0.001) (**Figures 6C,D**).

### Biased Functional Roles of Different Modes of Duplicated Genes

The conserved domains contained in protein sequence may be related to protein functions. Therefore, we identified the Pfam domains for protein sequences encoded by different modes of duplicated genes to resolve their biased functional roles (Supplementary Table 7). The proportion of different domains detected in each mode of duplicate genes was calculated. We also estimated the proportion of different domains in whole-genome proteins as the control. The first 10 domains with high frequency levels in each mode of gene duplication were selected for a comparative analysis (Supplementary Figure 7). The enriched domains for different modes of duplicate genes were biased. For WGD-derived genes, only two domains, PF00069.20 (protein kinase domain) and PF07714.12 (protein tyrosine kinase), were found to have slightly higher proportion than those found in total proteins. Protein kinases function in a multitude of cellular processes, including metabolism, transcription, signal transduction, cell cycle progression, cytoskeletal rearrangement and cell movement, apoptosis, and differentiation (Manning et al., 2002; Scheeff and Bourne, 2005). Therefore, the WGD-derived genes may play important roles in basal metabolism and biological regulation. Several domains involved in plant resistance and defense response, such as leucine rich repeat (PF12799.2, PF13855.1, PF00560.28, PF13504.1, and PF13516.1), cytochrome P450 (PF00067.17) and NB-ARC (PF00931.17) were overrepresented in TD- and PD-derived genes in a whole-genome protein comparison. RD-derived genes are enriched in pentatricopeptide repeat (PPR) domain (PF13812.1, PF13041.1, PF12854.2, and PF01535.15), F-box (PF00646.28), zinc-RING finger (PF14634.1), tetratricopeptide repeat (PF13428.1) domains. Interestingly, the pentatricopeptide repeat (PPR) domain was also overrepresented in DSD-derived genes. Prior studies revealed that PPR proteins play important roles in organellar gene expression, organelle (e.g., mitochondria and chloroplast) biogenesis and mRNA processing (Lurin et al., 2004; O’Toole et al., 2008). Thus, RD- and DSD-derived genes may be involved in the key metabolic processes in organelles. Notably, the ankyrin repeats domain (PF13857.1, PF13637.1, PF13606.1, PF12796.2, and PF00023.25) was overrepresented in DD-derived genes. Ankyrin repeat proteins are associated with plant organogenesis, male–female gamete recognition, and plant defense (Dong, 2004; Huang et al., 2006; Yu and Luan, 2010; Sharma and Pandey, 2015).

Moreover, we investigated the functional roles of different modes of duplicated genes through a GO enrichment analysis. The Gene Ontology Consortium classified all GO terms into three categories: MF, BP, and cellular component (CC) (Gene Ontology Consortium, 2004). First, we assigned pear genes into these three GO categories according to their GO annotations, and then we estimated the proportion of different GO categories detected in each mode of duplicate genes. Interestingly, the results showed that different modes of duplicated genes were biased toward particular categories (Supplementary Figure 8). WGD- and RD-derived genes were mainly involved in BP. TD-, PD-, and DSD-derived genes were enriched in the category MF. In particular, for PD-derived genes, the percentage in the MF category reached up to ∼70% and was higher than in the other modes. Sparklingly, RD- and DD-derived genes may have large contribution to the biosynthesis of cellular component with respect to the higher proportion of genes involved in the category cellular component. Secondly, we performed the GO enrichment analysis with strict statistical tests. The different GO terms appeared to be enriched in different modes of duplicate genes (Supplementary Table 8). The GO terms involved in “binding” and “regulation process,” such as transcription factor activity, sequence-specific DNA binding, zinc ion binding, regulation of macromolecule metabolic process, regulation of cellular metabolic process, and regulation of gene expression, were enriched in WGD-derived genes. The enriched GO terms in WGD-derived genes were also involved in the synthesis of some important cellular components, such as cytoplasmic part, macromolecular complex and intracellular organelle. For TD-derived genes, the overrepresented GO terms were related to “cell recognition” and “reproductive process,” such as recognition of pollen, single organism reproductive process, defense response and programmed cell death. The enriched GO terms in TD-derived genes are also related to “enzyme activity,” such as transferase, monooxygenase, electron carrier activity, hydrolase activity, and oxidoreductase activity levels. Like the TD-derived genes, the PD-derived genes were enriched for GO terms involved in immune response, programmed cell death, apoptotic process, response to stimulus, and defense response. Moreover, the enriched GO terms in PD-derived genes were also involved in “enzyme activity” and “binding,” such as transmembrane signaling receptor activity, monooxygenase activity, oxidoreductase activity, heme binding, and ATP binding. Only five GO terms, guanosine-containing compound metabolic process, extracellular matrix, nucleoside bisphosphate metabolic process, ribonucleoside bisphosphate metabolic process, and purine nucleoside bisphosphate metabolic process, were overrepresented (FDR corrected *P*-value < 0.05) in DSD-derived genes. However, we did not find significantly enriched GO terms in RD- and DD-derived genes after FDR correction. These results implied that different modes of duplicated genes have evolved toward to biased biological functions, which is fundamental for genome diversity and species survival.

### The Contribution of Gene Duplication to the Evolution of Gene Families Associated with Important Fruit Traits

**FIGURE 7:**
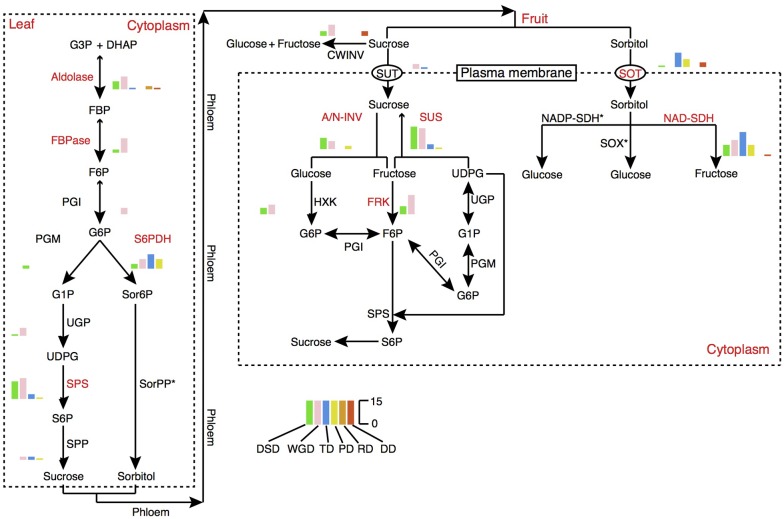
The expansion pattern of gene families involved in sugar metabolism pathways. The expanded gene families are marked in red color. The histogram indicates the numbers of different modes of duplicated genes. A/N-INV, alkaline/neutral invertase; CWINV, cell-wall invertase; DHAP, dihydroxyacetone phosphate; FBP, fructose 1,6-bisphosphate; FBPase, fructose-1,6-bisphosphatase; F6P, fructose 6-phosphate; FRK, fructokinase; G1P, glucose 1-phosphate; G3P, Glyceraldehyde 3-phosphate; G6P, Glucose 6-phosphate; HXK, hexokinase; NAD-SDH, NAD-sorbitol dehydrogenase; NADP-SDH, NADP-sorbitol dehydrogenase; PGI, phosphoglucose isomerase; PGM, phosphoglucomutase; Sor6P, sorbitol 6-phosphate; SorPP, sorbitol-6-phosphate phosphatase; S6P, sucrose 6-phosphate; S6PDH, sorbitol 6-phosphate dehydrogenase; SOT, sorbitol transporter; SPP, sucrose phosphate phosphatase; SPS, sucrose phosphate synthase; SUS, sucrose synthase; SUT, sucrose transporter; SOX, sorbitol oxidase; UDPG, UDP-glucose; UGP, UDP-glucose pyrophosphorylase.

Fruit quality and taste are largely influenced by the acidity and sugar levels. In pear fruit, the citric acid and malic acid are the two major components of organic acids. Sorbitol metabolism is the dominant feature of sugar-related metabolism in pear and other Rosaceae fruit crops. The 6-Phosphate dehydrogenase (S6PDH), sorbitol transporter (SOT), and NAD-sorbitol dehydrogenase (NAD-SDH) are closely related to the synthesis, degradation, and transportation of sorbitol. Therefore, we dissected the evolution and expansion patterns of the gene families involved in sugar and organic acid metabolism pathways in pear (**Figure 7**, Supplementary Figure 9, and Supplementary Table 9). The contributions of different modes of gene duplication to gene family expansion were also investigated. In total, 16 gene families were identified in the sugar metabolism pathway, and 9 gene families had expanded significantly relative to the gene families in Arabidopsis, including those encoding aldolase, fructose-1,6-bisphosphatase (FBPase), S6PDH, sucrose phosphate synthase (SPS), SOT, alkaline/neutral invertase (A/N-INV), sucrose synthase (SUS), fructokinase (FRK), and NAD-SDH. Interestingly, we observed that the gene family expansion found in the sucrose metabolism pathway (SPS, A/N-INV, SUS, FRK) was largely attributed to the WGD and DSD. In contrast, the single-gene duplications, including TD, PD and transposed (RD and DD) duplications, were the major contributors to the expansion of gene families in the sorbitol metabolism pathway (S6PDH, SOT, and NAD-SDH). The TDs and PDs were commonly younger and had higher evolutionary rates. In addition, 11 gene families involved in organic acid metabolism pathways were identified, and the 5, phosphoenolpyruvate carboxylase (PEPC), succinate dehydrogenase (SDH), NAD-malate dehydrogenase (NAD-MDH), aconitase (ACO), 2-oxoglutarate dehydrogenase (OGDH), that participated in the tricarboxylic acid (TCA) cycle have expanded in pear relative to Arabidopsis (Supplementary Figure 9). Moreover, we found that WGD event drove the expansion of TCA cycle-related gene families.

## Discussion

### Dramatic Genome Rearrangement and Local Gene Duplication Occurred Shortly after Polyploidization

The plant genome has evolved a robust tolerance to polyploidization and the following diploidization, which was accompanied by drastic genome changes, including extensive genome rearrangement, chromosomal number reduction and massive gene loss (Wendel, 2015). The remarkable plasticity of plant genomes can result in the short-term survival of polyploid and in long-term evolutionary significance by facilitating the transition from a polyploidy genome to a stable diploid-like form, which may eventually lead to the speciation (Leitch and Leitch, 2008; Dodsworth et al., 2015; Van de Peer et al., 2017). Intragenome conserved syntenic relationship can become obscure owing to the preferential gene loss and genome rearrangements over long time frames, resulting in remnants of a large number of ancestral syntenic gene pairs that deviated from the detection of synteny and collinearity (Tang et al., 2008a). These deviating syntenic pairs may be an import resource of dispersed genes, although the single gene transposition and/or relocation may account for the invasiveness of dispersed duplicates (Wang et al., 2012b, 2016). In the present study, substantial DSDs were detected in the pear genome, mirroring the considerable chromosomal rearrangements that occurred after WGDs. Additionally, some chromosomal regions with a low density of WGD-derived genes often showed a high density of DSD-derived genes. This suggested that the ancestral gene order had been largely reshuffled during the rediploidization process. Two genome-wide duplication events have been detected in the pear genome: the ancient WGD event corresponding to the core-eudicot γ triplication (*K*_s_ = 1.5–1.8) that occurred ∼140 million years ago (MYA) and the recent WGD (*K*_s_ = 0.15–0.3), which was dated to 30–45 MYA (Wu et al., 2013). Indeed, we observed two corresponding peaks in the distribution of *K*_s_ values for WGD-, RD-, DD-, and DSD-derived pairs. Furthermore, we found that the two *K*_s_ peaks for WGD-, RD-, DD-, and DSD-derived pairs emerged at similar *K*_s_ regions, suggesting that large-scale transposed and dispersed gene duplication occurred very shortly after genome duplication. Substantial numbers of dispersed duplicates may have been generated by large-scale gene relocations that occurred shortly after the core-eudicot γ triplication, and they play important roles in the diversification of core eudicots (Wang et al., 2016). Therefore, the contribution of dispersed duplicates to the biological innovation needs to be further investigated. In addition, the high frequency of TDs or PDs was detected in some chromosomal regions having a high density of WGD-derived genes. Such a tendency was observed more obviously for the genomic density between WGD- and TD-derived genes. Only one *K*_s_ peak was observed for TD- or PD-derived pairs, and this overlapped with the *K*_s_ peak for the recent WGD. This suggested that the more recent origination of TD- and PD-derived genes that may have been simulated by polyploidization-diploidization and other factors, such as environment stimuli. TDs and PDs are important for stress responses and adaption to changing environments in plant (Hanada et al., 2008; Woodhouse and Freeling, 2009). In the carnivorous plant-*Utricularia gibba*, TDs are major contributors to the expansion of gene families associated with prey trapping and processing (Lan et al., 2017). In *Thellungiella parvula*, the preferential expansion of genes involved in stress defense responses was attributed to TDs and important for the adaptation to extreme environments, such as saline, resource-poor habitats (Dassanayake et al., 2011). Additionally, over 44.0 and 51.9% of the NBS-encoding resistance genes have experienced tandem duplication in *Brassica oleracea* and *B. rapa*, respectively (Liu et al., 2014). These results collectively suggested that the expansion of TD- and PD-derived genes following WGD would greatly contribute to the robust resistance robustness against to abiotic and biotic stresses in plants.

### Widespread Sequence, Expression and Regulatory Divergence Occurred Following Gene or Genome Duplication

Different modes of duplicated genes showed distinct evolutionary patterns in protein-coding region. Transposed genes (RD or DD) and DSDs that are preserved in pear genome present more extensive divergence in non-synonymous substitutions per site than other modes of duplicated genes, suggesting their prominent roles in contribution to evolutionary novelty. PDs and TDs had relatively high *K*_a_/*K*_s_ ratios but relatively small *K*_s_ values (younger age), implying that they have experienced more rapid functional divergence than other gene classes and also suggesting the important roles of positive selection in the early stage of duplicate gene retention (Shiu et al., 2006; Ren et al., 2014; Cardoso-Moreira et al., 2016). In contrast, WGD-derived pairs have relatively small *K*_a_ values and low *K*_a_/*K*_s_ ratios, suggesting that they evolved under strong purifying selection over a long time.

In parallel with sequence divergence, different modes of duplicated pairs in pear have extensively diverged in expression, especially for RD-, DD-, and DSD-derived pairs, in which over 70% gene pairs have experienced expression divergence, respectively. This observation is consistent with previous studies in which the expression divergence between duplicate genes has been widely delineated (Gu et al., 2002; Blanc and Wolfe, 2004; Li et al., 2005). In Arabidopsis, 57% of young duplicate pairs (∼35 MYA) and 73% of old duplicate pairs (50–60 MYA) have diverged in expression (Blanc and Wolfe, 2004). In the cotton-D genome (*Gossypium raimondii*), over 85% of the gene pairs that survived from a recent genome duplication event (∼60 MYA) exhibit differential expression (Renny-Byfield et al., 2014). Thus, the expression divergence of ancient paralog pairs is important for the preservation of gene duplicates over the long-term evolution (Li et al., 2005; Ha et al., 2007; Huerta-Cepas et al., 2011).

Moreover, the different classes of duplicated pairs in pear have dramatically diverged in their promoter regions, especially for RD-, DD-, and DSD-derived pairs, in which over 80% gene pairs have experienced promoter divergence, respectively. The divergence in the promoter region appears to be more extensive than in spatiotemporal expression. However, a clear relationship between expression divergence and promoter divergence was not detected in this study. This result is similar to a previous study in which a weak correlation between expression divergence and promoter regulatory-motif divergence was found (Zhang et al., 2004). One possible explanation for this association is that gene expression can be regulated by many other *trans*-acting factors in complicated gene regulatory networks (Wray et al., 2003; Yvert et al., 2003).

### Different Modes of Duplicate Genes Exhibited Distinct Functional Roles

In this study, we found evidence for the differential functional roles of different classes of duplicated genes. WGD-derived genes played prominent roles in the expansion of gene families participating in the regulatory and synthetic processes of some important cellular components, supporting the observations of previous studies in which the increase in gene families involved in transcriptional regulation was largely attributed to the WGD (or defined as polyploidization) (Seoighe and Gehring, 2004; Maere et al., 2005; Shiu et al., 2005). The roles of ancient polyploidization events in adapting to stressful environmental conditions have been suggested in prior studies (Fawcett et al., 2009; Mable et al., 2011; Vanneste et al., 2014). In plants, a wave of WGDs have been detected around the Cretaceous–Paleogene (K–Pg) boundary, which was a time of environmental upheaval that lead to the mass extinction of plants and animals (Vanneste et al., 2014). A WGD can be resulted in a rapid increase in the genes involved in transcriptional regulation and cellular components synthesis, and thus reduce the risk of extinction under extreme environmental conditions. In this study, TDs and PDs were collectively enriched in the GO terms involved in defense response, programmed cell death, apoptotic process, monooxygenase activity, and oxidoreductase activity, suggesting important roles in removing damaged cell or tissues and preventing pathogen infection. Moreover, the TDs were also involved in the recognition of pollen and single organism reproductive processes, suggesting their potential roles in the process of self-incompatibility. In addition, the PDs were also related to immune response and stimulus or stress responses, implying roles in plant adaptation. For instance, TDs may play an important role in the expansion of some transcription factor families (Lehti-Shiu et al., 2017). Over 44.0 and 51.9% of the NBS-encoding resistance genes have undergone tandem duplications in *B. oleracea* and *B. rapa*, respectively (Liu et al., 2014). Thus, the increasing number of TD- and PD-derived genes after WGD can enhance the level of plant resistance against to abiotic and biotic stresses.

### The Roles of Gene Dosage Balance in the Retention of WGD-Derived Duplicates

Gene dosage balance has been suggested an important driving force in maintaining WGD genes and increasing morphological complexity (Freeling and Thomas, 2006; Birchler and Veitia, 2007). Under this model, dosage or stoichiometric relationships are balanced immediately after genome duplication events, and the mutation/loss of one copy of a duplicated pair will result in the decreased fitness and the phenotypic variation (Birchler and Veitia, 2012). The purifying selection driven by dosage-balance constraints can eliminate the deleterious mutations and protect both gene copies from functional divergence. In this study, the duplicates derived from WGD exhibited lower *K*_a_/*K*_s_ ratios, weaker expression divergence, and appear to be more conserved than other modes of gene duplication. This result can be largely explained by the dosage-balance hypothesis, which suggests that purifying selection maintains the ancestral functions of two gene copies and prevents the divergence of duplicate genes to maintain the stoichiometric balance. In addition, the duplicated genes involved in signal transduction, transcriptional regulation, and macromolecular complexes tend to be preferentially retained after WGD, which can be attributed to the dosage constraint (Blanc and Wolfe, 2004; Paterson et al., 2006; Freeling, 2009; Conant et al., 2014). Here, the gene dosage-balance model is further supported by the enrichment in GO terms for regulatory and metabolic genes among the WGD duplicates detected in the pear genome.

In summary, we identified the different modes of duplicated genes in pear genome. Widespread sequence, expression and regulatory divergence have occurred between duplicated genes over 30–45 million years of evolution after the recent WGD event in pear. Different modes of duplicate genes exhibited biased functional roles. Moreover, we observed that the TDs and PDs largely accounted for the extensive expansion of gene families involved in the sorbitol metabolism pathway, while WGD and/or DSD are responsible for the gene family expansion in the sucrose and TCA cycle-related metabolism pathways in pear. The results from this study enhance our understanding of the evolution and retention mechanisms of duplicated genes.

## Author Contributions

SZ and XQ conceived and designed the experiments. XQ carried out the experimental design, data analysis, and drafted the manuscript. HY, LL, and RW contributed analytic tools and Perl scripts. JyW and JW contributed advice. SZ managed the research and experiments.

## Conflict of Interest Statement

The authors declare that the research was conducted in the absence of any commercial or financial relationships that could be construed as a potential conflict of interest.

## Footnotes

1 http://peargenome.njau.edu.cn/

2 https://www.ncbi.nlm.nih.gov/bioproject/PRJNA246160

3 http://www.rosaceae.org/

4 http://www.phytozome.net/

5 http://chibba.agtec.uga.edu/duplication/

6 https://www.ncbi.nlm.nih.gov/sra

7 http://pfam.xfam.org/

8 http://peargenome.njau.edu.cn/

9 https://phytozome.jgi.doe.gov/pz/portal.html
