# Supplementary material for: Different Modes of Gene Duplication Show Divergent Evolutionary Patterns and Contribute Differently to the Expansion of Gene Families Involved in Important Fruit Traits in Pear (Pyrus bretschneideri)
Source: Front Plant Sci. 2018 Feb 13;9:161. doi: 10.3389/fpls.2018.00161 (PMC5816897; doi:10.3389/fpls.2018.00161)
Supplement: Supplementary file 2 [file Image_1.PDF]

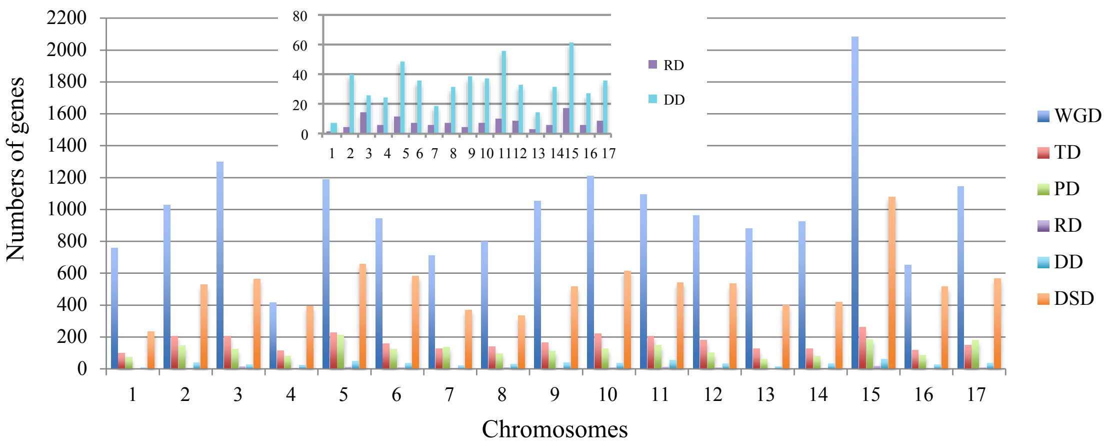

Supplemental Figure 1. The number of different modes of duplicate genes on each of pear 17 chromosomes.

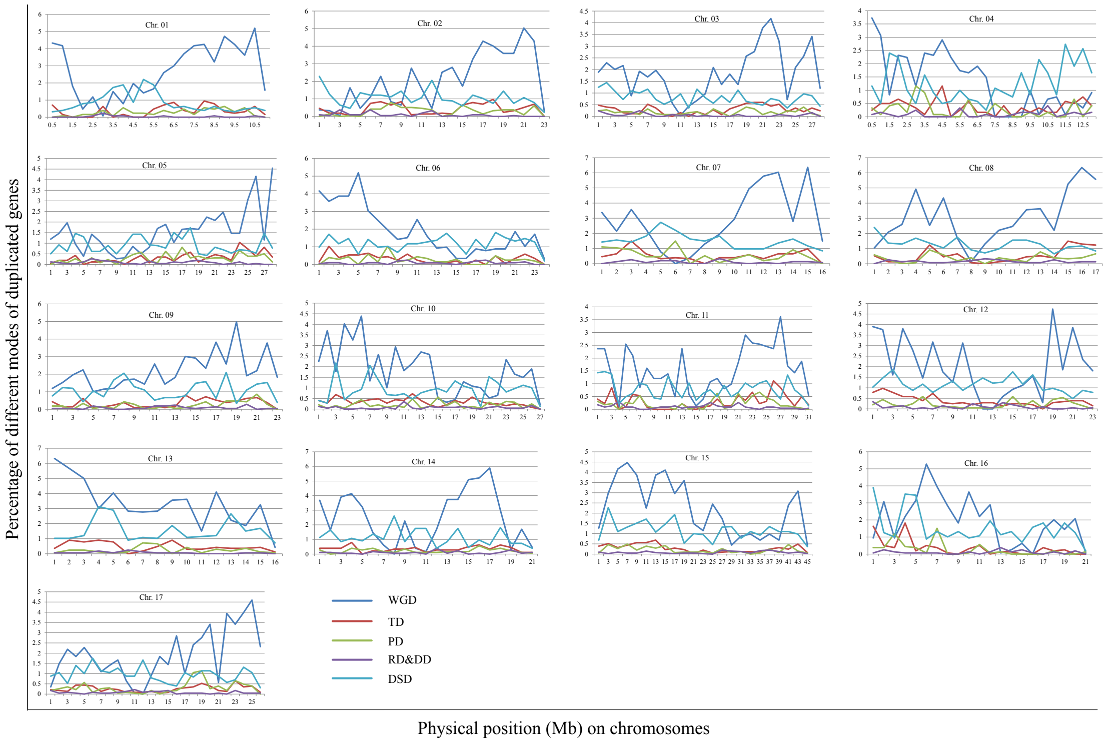

Supplemental Figure 2. The distribution of different modes of duplicate genes along each of pear 17 chromosomes.

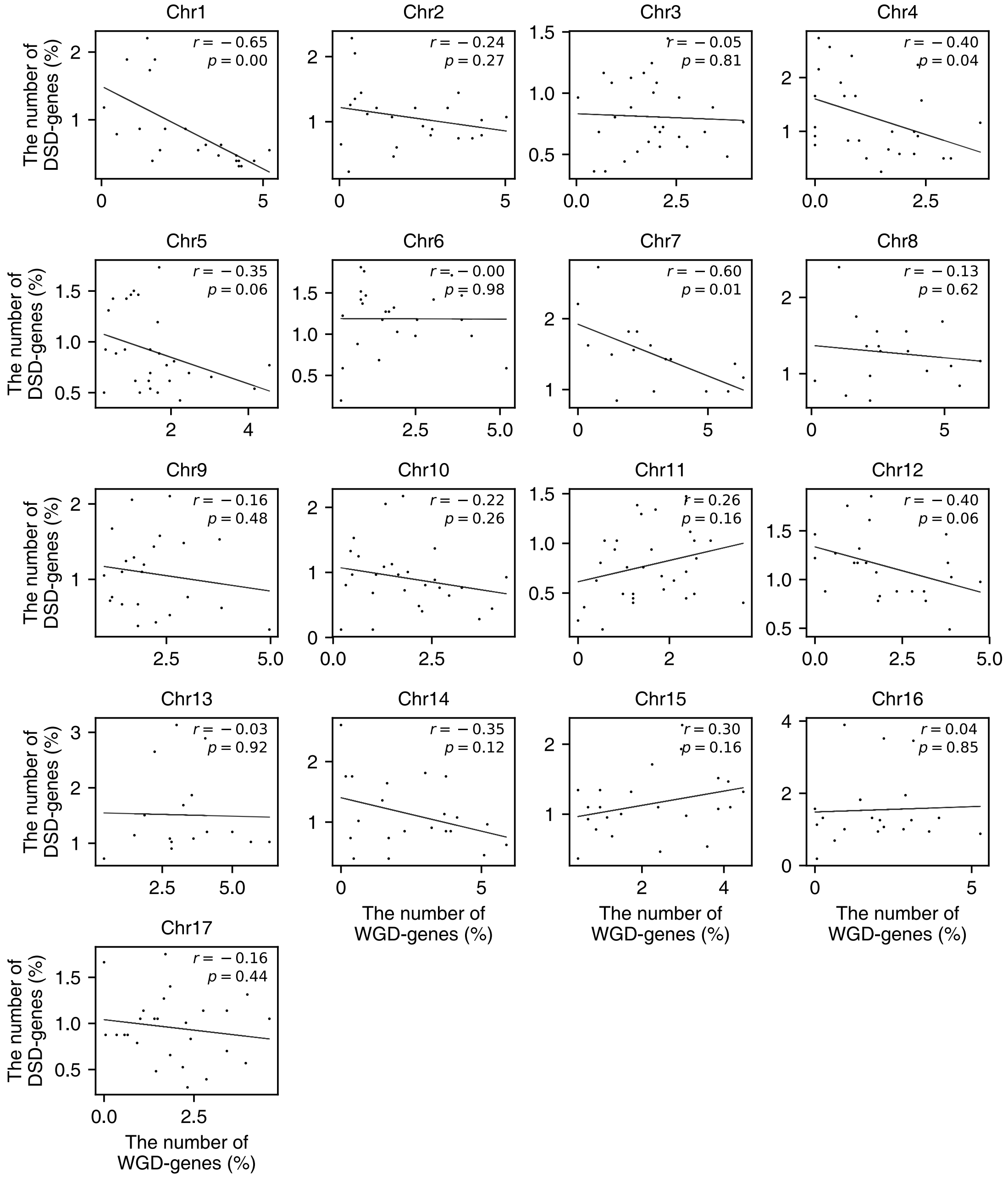

Supplemental Figure 3. The correlation of genomic density between WGD- and DSD-genes in pear 17 chromosomes. We transformed the number of WGD- or DSD-genes in each chromosome into a relative number. The relative number is computed as the absolute number of WGD- or DSD-genes divided by the total number of genes in each chromosome.

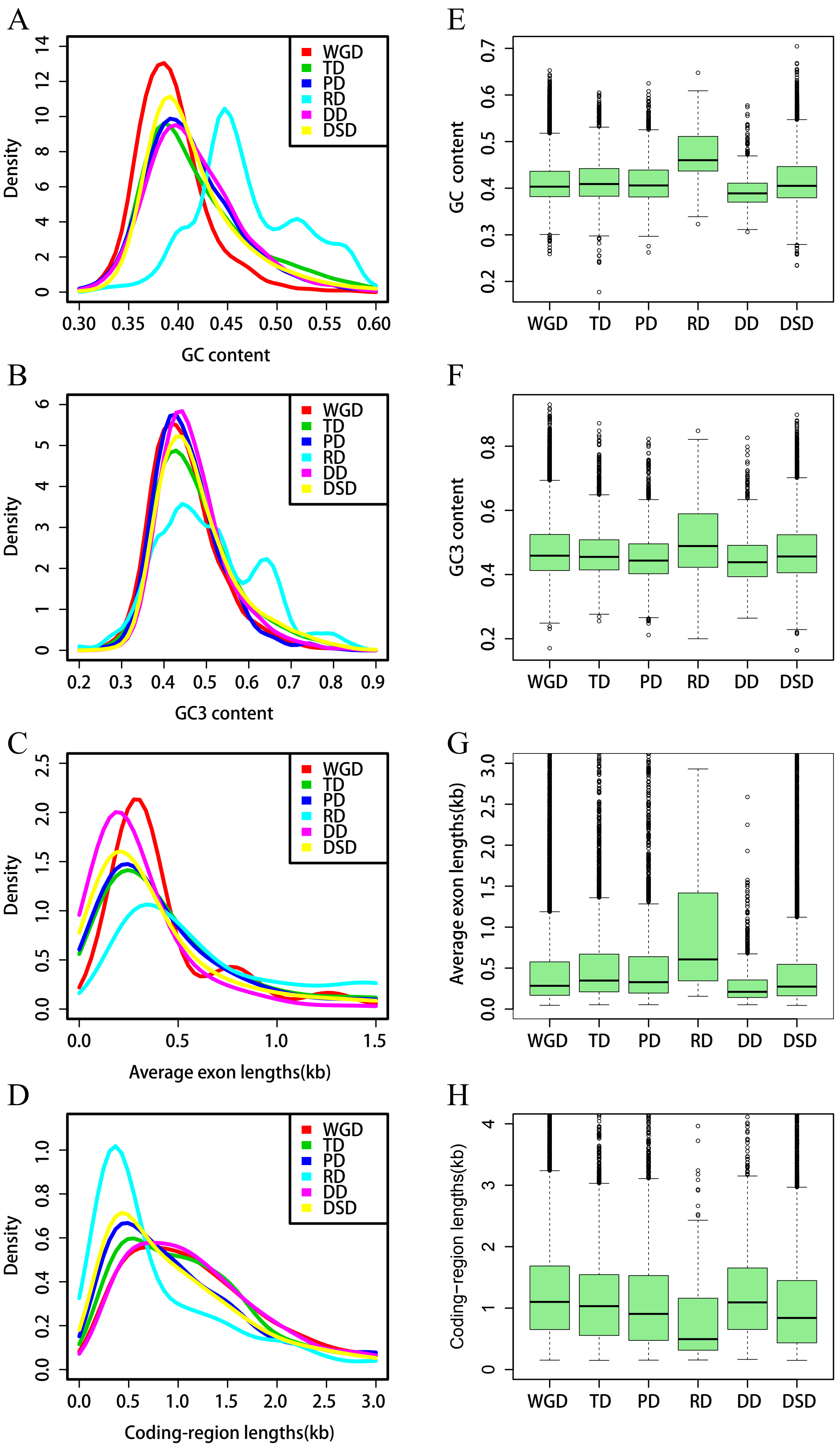
A
Density
GC content
0.30 0.35 0.40 0.45 0.50 0.55 0.60
0 2 4 6 8 10 12 14
WGD
TD
PD
RD
DD
DSD
B
Density
GC3 content
0.2 0.3 0.4 0.5 0.6 0.7 0.8 0.9
0 1 2 3 4 5 6
C
Density
Average exon lengths(kb)
0.0 0.5 1.0 1.5
0.0 0.5 1.0 1.5 2.0 2.5
D
Density
Coding-region lengths(kb)
0.0 0.5 1.0 1.5 2.0 2.5 3.0
0.0 0.2 0.4 0.6 0.8 1.0
E
GC content
0.2 0.3 0.4 0.5 0.6 0.7
WGD TD PD RD DD DSD
F
GC3 content
0.2 0.4 0.6 0.8
WGD TD PD RD DD DSD
G
Average exon lengths(kb)
0.0 0.5 1.0 1.5 2.0 2.5 3.0
WGD TD PD RD DD DSD
H
Coding-region lengths(kb)
0 1 2 3 4
WGD TD PD RD DD DSD

Supplemental Figure 4. Gene features of different modes of duplicate genes. (A, E) GC content; (B, F) GC3 content; (C, G) Average exon lengths (kb); (D, H) Coding-region lengths (kb).

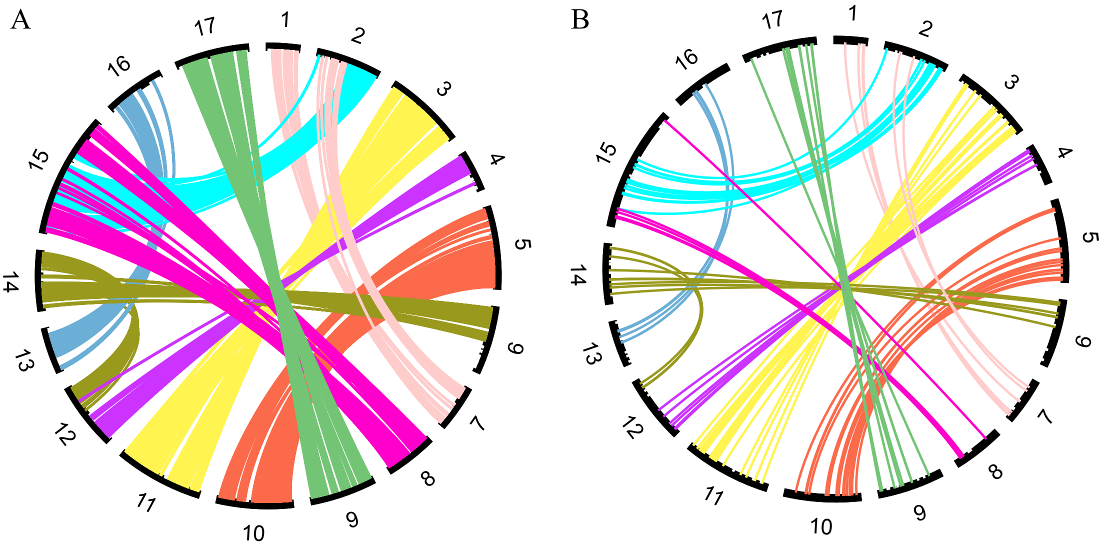

Supplemental Figure 5. Gene conversion detected in WGD-pairs. (A) The WGD-pairs derived from recent genome duplication. The homologous chromosomal region is linked by colored lines. (B) Converted WGD-pairs and their distribution on syntenic blocks.

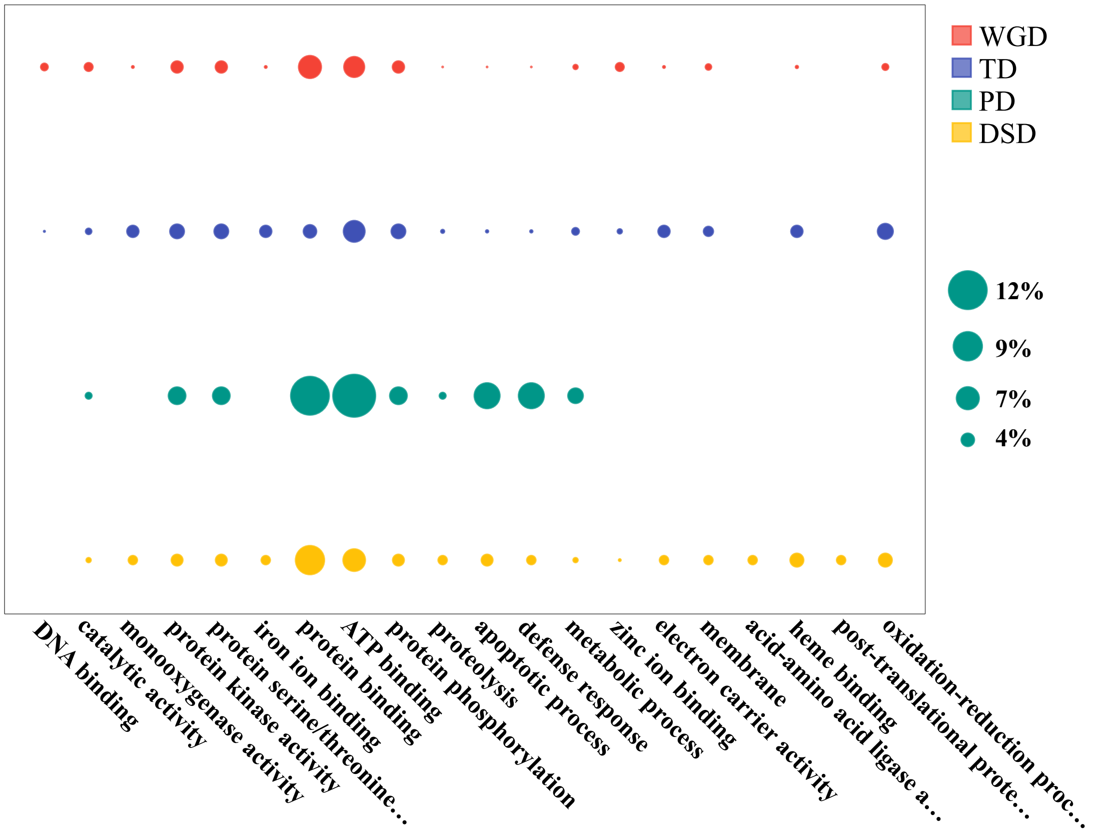

Supplemental Figure 6. GO analysis for the duplicate genes influenced by gene conversion. The colored circle indicates the proportion of top GO terms in each class of duplicate genes influenced by gene conversion. The bigger circle indicates a higher frequency of occurrence of one GO term in each mode of gene duplication.

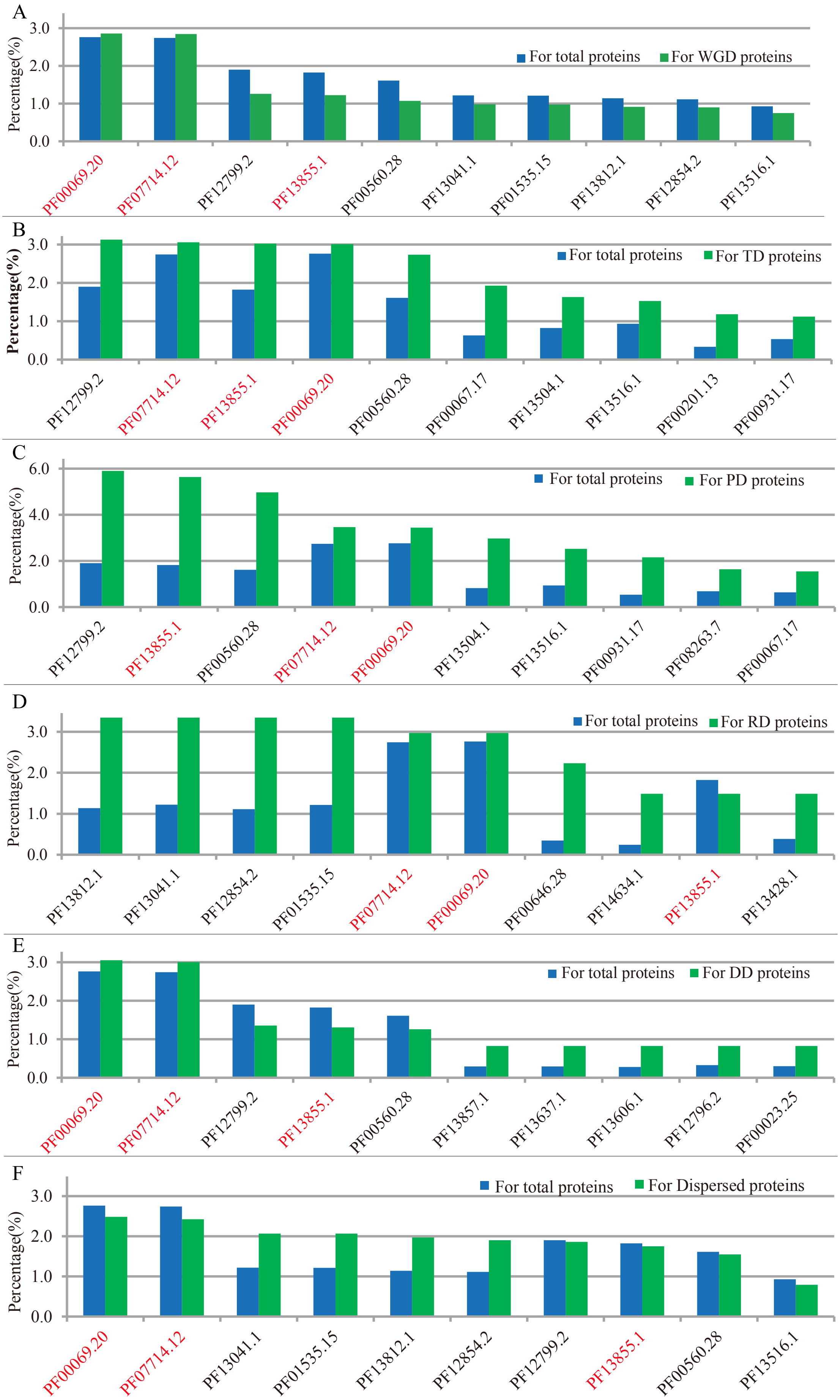
A
Percentage(%)
3.0
2.0
1.0
0.0
For total proteins
For WGD proteins
PF00069.20
PF07714.12
PF12799.2
PF13855.1
PF00560.28
PF13041.1
PF01535.15
PF13812.1
PF12854.2
PF13516.1
B
Percentage(%)
3.0
2.0
1.0
0.0
For total proteins
For TD proteins
PF12799.2
PF07714.12
PF13855.1
PF00069.20
PF00560.28
PF00067.17
PF13504.1
PF13516.1
PF00201.13
PF00931.17
C
Percentage(%)
6.0
4.0
2.0
0.0
For total proteins
For PD proteins
PF12799.2
PF13855.1
PF00560.28
PF07714.12
PF00069.20
PF13504.1
PF13516.1
PF00931.17
PF08263.7
PF00067.17
D
Percentage(%)
3.0
2.0
1.0
0.0
For total proteins
For RD proteins
PF13812.1
PF13041.1
PF12854.2
PF01535.15
PF07714.12
PF00069.20
PF00646.28
PF14634.1
PF13855.1
PF13428.1
E
Percentage(%)
3.0
2.0
1.0
0.0
For total proteins
For DD proteins
PF00069.20
PF07714.12
PF12799.2
PF13855.1
PF00560.28
PF13857.1
PF13637.1
PF13606.1
PF12796.2
PF00023.25
F
Percentage(%)
3.0
2.0
1.0
0.0
For total proteins
For Dispersed proteins
PF00069.20
PF07714.12
PF13041.1
PF01535.15
PF13812.1
PF12854.2
PF12799.2
PF13855.1
PF00560.28
PF13516.1

Supplemental Figure 7. The proportion of different Pfam domains detected in each class of duplicate genes. The top 10 domains with high frequency in each class of duplicate genes were showed. The proportion of different domains in whole-genome proteins was used as the control.

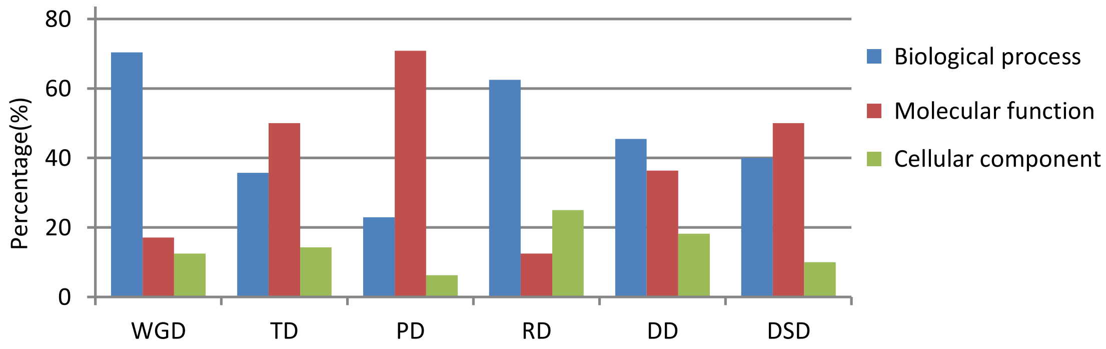

Supplemental Figure 8. The proportion of three top GO categories detected in each class of duplicate genes. All GO terms were classified into three top categories by Gene Ontology Consortium: molecular function (MF), biological process (BP), and cellular component (CC).

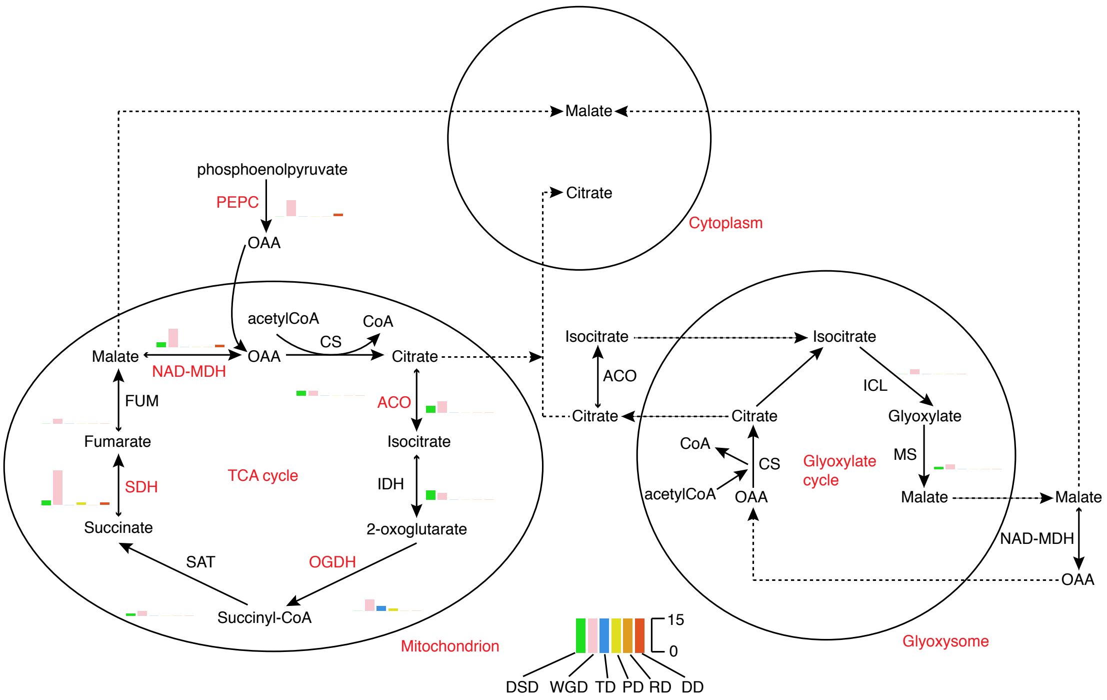

Supplemental Figure 9. The expansion pattern of gene families involved in organic acid metabolism pathways. The expanded gene families were marked in red color. The histogram indicates the numbers of different modes of duplicated genes. ACO, aconitase; CS, citrate synthase; FUM, fumarase; ICL, isocitrate lyase; IDH: isocitrate dehydrogenase; MS, malate synthase; NAD-MDH: NAD-malate dehydrogenase; OAA: Oxaloacetic acid; OGDH, 2-oxoglutarate dehydrogenase; PEPC, phosphoenolpyruvate carboxylase; SAT, succinate thiokinase; SDH, succinate dehydrogenase.
